# R Scragg's and JD Slutyer's “Is There Proof of Extraskeletal Benefits From Vitamin D Supplementation From Recent Mega Trials of Vitamin D?”

**DOI:** 10.1002/jbm4.10491

**Published:** 2021-03-30

**Authors:** Barbara J Boucher, William B Grant

**Affiliations:** ^1^ Blizard Institute, Barts & The London School of Medicine & Dentistry, Queen Mary University of London London UK; ^2^ Sunlight, Nutrition, and Health Research Center San Francisco CA USA

## Peer Review

The peer review history for this article is available at https://publons.com/publon/10.1002/jbm4.10491.

**To The Editor**

From an analysis of the trial findings of vitamin D supplementation with at least 2000 recruits carried out between 2009 and 2020, Scragg and Sluyter found that benefits in bone health were produced mainly in initially deficient subjects,^(^ ^1^ ^)^ as one might expect. Therefore, they concluded that population‐based measures such as food fortification would be a better approach to correcting population deficiency than screening to identity deficient subjects. Food fortification has already been shown to be effective when used together with the encouragement of high‐risk group supplementation, for example in Finland, for correcting deficiency.^(^ ^2^ ^)^ Only four of the seven trials that met the authors stated criteria had reported their findings of both improved bone health together with some improvement in both vascular and lung function with supplementation, though they showed no consistent reductions in nonskeletal health outcome risks other than in cancer mortality. The authors suggest that the results from the four as‐yet unpublished randomized control trials and further outcome analyses from the three published trials “can be expected to clarify the role of vitamin D supplementation for reducing nonskeletal disease.” However, they did not discuss what specific data analyses would be the most likely to reveal any such health benefits. Because many aspects of the biology of vitamin D can reduce the ability of trial data to demonstrate health benefits, it is hoped that they can be allowed for when that further trial data comes to be analyzed. For example, the nonlinearity of the effects of vitamin D means that increasing intakes produce S‐shaped curves, both for the increases in serum 25(OH)D with intake and for the effects produced by increases in serum 25(OH)D. Thus, the effects of supplementation are minimal if deficient 25(OH)D values are not raised onto the steep slope of the S and with the supplementation of replete subjects whose response data are already on the upper plateau of the S.^(^ ^3^ ^)^

Furthermore, effect thresholds for vitamin D's effects on different functions and disorders vary with serum 25(OH)D concentrations and not with intact vitamin D_3_ intakes.^(^ ^4^ ^)^ Thus, trial analyses should examine basal 25(OH)D values so that outcomes can be examined in subjects whose deficiency was corrected and should also examine outcomes by achieved 25(OH)D values.^(^ ^5^ ^)^ Also, because few 25(OH)D thresholds have been identified, they should look for possible thresholds at and above which the desired outcome appears and check whether enough subjects achieved such thresholds for relevant subgroup analyses to be valid. Already known serum 25(OH)D threshold effects include, for example, 50 nmol/l for bone outcomes but 80–100 nmol/l for reducing abnormal insulin resistance.^(^ ^6^ ^)^ Because raised insulin resistance is a major cause of type 2 diabetes mellitus (T2DM) development, reducing insulin resistance is especially important for reducing T2DM risks in those with prediabetes.^(^ ^7^ ^)^ This concept is confirmed by the recent D2d data reanalysis showing that T2DM risks were reduced by up to 70% in those achieving average intratrial 25(OH)D values of 100 nmol/l or more, although it is of interest that those values were only achieved on intakes of 4000 IU/day and not on 3200 IU/day or less.^(^ ^8^ ^)^ These findings imply that many trials may well not have achieved serum 25(OH)D effect threshold values for the outcome(s) of interest because the supplemental doses were too low, as the authors note. Hence, this situation provides an important reason for measuring achieved 25(OH)D values.

Although most randomized clinical trials targeted at the risks of cardiovascular disease (CVD) have not shown risk reductions based on the problems of trial design and the life‐long natural history of CVD development versus trial duration, CVD risk factors are commonly less abnormal with higher D status observationally, implying that vascular health benefits should appear with suitably high vitamin D status over the lifespan. Acute events, however, may well be reduced by adequate supplementation because the plaque disruption that leads to occlusive clot formation follows inflammatory changes with infiltration by macrophages that secrete tissue‐destructive matrix metalloproteinases (especially MMPs‐2/9), whereas inflammation is well known to be reduced by improving vitamin D status and MMP‐2/9 secretion is reduced by vitamin D supplementation,^(^ ^9^ ^)^ though the necessary 25(OH)D thresholds are unknown. In addition, concomitant supplemental calcium intakes are often not allowed for in trial analyses though increased supplemental, but not dietary intakes may increase CVD risks, at least in women.^(^ ^10^ ^)^

Though there are other problems that can upset trial data analyses, including 25(OH)D assay variability and genetic variations affecting serum 25(OH)D values, one can predict that when measurements of baseline and achieved serum 25(OH)D are used in health‐outcome analyses of trial data the findings will consistently prove to be more definitive for confirming or disproving the causality of vitamin D status for nonskeletal health outcomes than the results of health‐outcome analysis by supplemental vitamin D alone.

## Conflict of Interest

Barbara J Boucher has no conflicts of interest to declare. William B Grant receives funding from Bio‐Tech Pharmacal.

## References

[1] Scragg R , Sluyter JD . Is there proof of extraskeletal benefits from vitamin D supplementation from recent mega trials of vitamin D? JMBR Plus. 2020;23:e10459. 10.1002/jbm4.10459 PMC7839821 33553994

[2] Jaaskelainen T , Itkonen ST , Lundqvist A , et al. The positive impact of general vitamin D food fortification policy on vitamin D status in a representative adult Finnish population: evidence from an 11‐y follow‐up based on standardized 25‐hydroxyvitamin D data. Am J Clin Nutr. 2017;105(6):1512‐1520. 28490516 10.3945/ajcn.116.151415

[3] Heaney RP . Guidelines for optimizing design and analysis of clinical studies of nutrient effects. Nutr Rev. 2014;72(1):48‐54. 10.1111/nure.12090 24330136

[4] Scragg R . Emerging evidence of thresholds for beneficial effects from vitamin D supplementation. Nutrients. 2018;10(5):561. 10.3390/nu10050561 PMC5986441 29751504

[5] Grant WB , Boucher BJ , Bhattoa HP , Lahore H . Why vitamin D clinical trials should be based on 25‐hydroxyvitamin D concentrations. J Steroid Biochem Mol Biol. 2018;177:266‐269. 28842142 10.1016/j.jsbmb.2017.08.009

[6] von Hurst PR , Stonehouse W , Coad J . Vitamin D supplementation reduces insulin resistance in South Asian women living in New Zealand who are insulin resistant and vitamin D deficient—a randomised, placebo‐controlled trial. Br J Nutr. 2010;103(4):549‐555. 19781131 10.1017/S0007114509992017

[7] Boucher BJ . Can vitamin D supplementation reduce insulin resistance and hence the risks of type 2 diabetes? J Diabetes Clin Res. 2020;2(1):1‐8.

[8] Dawson‐Hughes B , Staten MA , Knowler WC , et al. Intratrial exposure to vitamin D and new‐onset diabetes among adults with prediabetes: a secondary analysis from the vitamin D and type 2 diabetes (D2d) study. Diabetes Care. 2020;43(12):2916‐2922. 33020052 10.2337/dc20-1765 PMC7770274

[9] Timms PM , Mannan N , Hitman GA , et al. Circulating MMP9, vitamin D and variation in the TIMP‐1 response with VDR genotype: mechanisms for inflammatory damage in chronic disorders? QJM. 2002;95(12):787‐796. 12454321 10.1093/qjmed/95.12.787

[10] Boucher BJ . Calcium supplements may increase the risk of cardiovascular events in postmenopausal women. Evid Based Med. 2012;17(1):16‐17. 22028370 10.1136/ebm.2011.100113

